# Alveolar bone remodeling during maxillary incisor intrusion and retraction

**DOI:** 10.1186/s40510-019-0300-2

**Published:** 2019-12-23

**Authors:** Seok Yoon Hong, Jeong Won Shin, Christine Hong, Vania Chan, Un-Bong Baik, Young Ho Kim, Hwa Sung Chae

**Affiliations:** 1 Department of Orthodontics, Institute of Oral Health Science, Ajou University School of Medicine, Suwon, South Korea; 2 Division of Orthodontics. School of Dentistry, University of California, San Francisco, CA USA; 3 School of Dentistry, University of California, Los Angeles, CA USA

**Keywords:** Alveolar bone remodeling, Intrusion, Retraction, Maxillary incisors, Prediction factors, Malocclusion

## Abstract

**Background:**

Maxillary incisor protrusion is a prevalent dental deformity and is often treated by upper incisor intrusion and retraction. The mechanical loading triggers the resorption and apposition of the bone. Alveolar bone remodeling is expected to follow orthodontic tooth movement in a one-to-one relationship. However, in many cases, the outcomes are different. Alveolar bone might still remain thick causing lip protrusion and other aesthetic problems after treatment. Additional corrective procedures such as alveoloplasty. On the other hand, if the labial bone becomes too thin, periodontal problems like gingival recession might occur. The unpredictability of the treatment result and the risk of requiring corrective procedures pose significant challenges to both the providers and patients. The aim of this study is to determine factors that can help to predict the alveolar bone reaction before maxillary incisor intrusion and retraction.

**Methods:**

The cohort included 34 female patients (mean age 25.8 years) who were diagnosed with skeletal class II malocclusion with upper incisor protrusion. These patients underwent extraction and orthodontic treatment with upper incisor intrusion and retraction. Lateral cephalograms at pre-treatment and post-treatment were taken. Linear and angular measurements were analyzed to evaluate the alveolar bone changes based on initial conditions.

**Results:**

The study found that the relative change, calculated as change in alveolar bone thickness after treatment divided by the initial alveolar thickness, was inversely correlated with the initial thickness. There was a significant increase of labial alveolar bone thickness at 9-mm apical from cementoenamel junction (B3) (*P* < 0.05) but no statistically significant change in the thickness at other levels. In addition, the change in angulation between the incisor and alveolar bone was inversely correlated with several initial angulations: between the initial palatal plane and upper incisor angle, between the initial palatal plane and upper incisor labial surface angle, and between the initial palatal plane and bone labial surface angle. On the other hand, the change in labial bone thickness was neither significantly correlated with the initial thickness nor significantly correlated to the amount of retraction.

**Conclusion:**

The unpredictability of alveolar bone remodeling after upper incisor intrusion and retraction poses significant challenges to treatment planning and patient experience. The study showed that the initial angulation between the incisor and alveolar bone is correlated with the change in angulation after treatment, the initial thickness of the alveolar bone was correlated with the relative change of the alveolar bone thickness (defined as change in thickness after treatment divided by its initial thickness), and the amount of intrusion was correlated with the alveolar bone thickness change at 9-mm apical from the cementoenamel junction after treatment. The results of the present study also revealed that the change in labial alveolar bone thickness was neither significantly correlated with the initial thickness nor significantly correlated to the amount of retraction.

## Background

Maxillary incisor protrusion is often orthodontically treated, most commonly by extracting premolars and retracting anterior teeth with maximum anchorage. It is often thought that alveolar bone remodeling follows orthodontic tooth movement. Retracted teeth move through the alveolar bone, causing bone remodeling to occur with potential change in bone density and thickness to adapt to its new position [1–3]. Premolar extraction and orthodontic correction have been shown to be very effective in reducing dental protrusion in many studies [4].

Nonetheless, it is found that the reaction of the alveolar bone surrounding the maxillary incisors does not always react to the tooth movement as expected. It is still unclear if the direction and amount of movement are always in synchronization for all anteroposterior, vertical and transversal directions [5]. Furthermore, case reports demonstrated that dehiscence and fenestration were observed despite improvement of teeth protrusion after orthodontic treatment [6]. In some cases, even after the retraction of incisors, a much thicker alveolar bone was left. This results in remaining lip protrusion and other aesthetic problems and usually requires additional periodontal surgery, such as alveoloplasty [7, 8]. On the other hand, if the labial bone is thin, a thin gingival biotype is expected and can cause periodontal problems like gingival recession [9].

The unsatisfactory results from upper incisor intrusion and retraction treatment are still not completely understood. It is difficult to predict the efficacy of a treatment because there are many factors including an individual’s initial dental conditions, the optimal amount of orthodontic force, and inclination of the intrusion and retraction, which can potentially influence the treatment and affect the results in various degrees.

The purpose of this study is to determine factors that can help to predict the alveolar bone reaction before maxillary incisor intrusion and retraction. The results can provide more insight into safer and more reliable orthodontic treatment.

## Material and methods

This study was approved by the institutional review board (IRB) of the Ajou University Hospital (IRB No: AJIRB-MED-MDB-18-295). Thirty-four Korean female patients (mean age 25.8 years, from 14 to 49 years) who underwent extraction and orthodontic treatment with upper incisor intrusion and retraction were examined. Inclusion criteria was the diagnosis of skeletal class II malocclusion with upper incisor protrusion, according to Steiner’s analysis: Angle between the lines NA and NB (ANB) > 2°, linear measure between the most vestibular point of the upper incisor and the NA line (upper central incisor, U1 to N-A) > 4 mm and minor crowding in the maxillary arch < 3 mm. Exclusion criteria were (1) patients with a medical history related to bone metabolism problems, (2) patients taking anti-inflammatory drugs during treatment or within 6 months before treatment, (3) patients with periodontal or gingival diseases at the beginning of orthodontic treatment, and (4) patients with a history of upper incisor trauma.

All the patients were treated by one clinician. In all cases, 0.022-in. MBT brackets were bonded, followed by sequential wire change adopting 0.016-in. nickel titanium, 0.018 in. × 0.025 in. Bioforce (Sirona, USA) and 0.019 × 0.025 stainless steel for space closing. Both sliding and loop mechanic were used. Mostly, the maxillary 1st premolars were extracted, and the maximum anchorage was prepared. The treatment duration to retract incisors was 6 to 9 months to retract incisors. To conduct intrusion and retraction of the maxillary incisors, since the center of resistance of the maxillary anterior six teeth is closely located between upper lateral incisors and canines [10, 11], TADs (temporary anchorage devices) were inserted there in most cases unless those TADs failed.

**Fig. 1:**
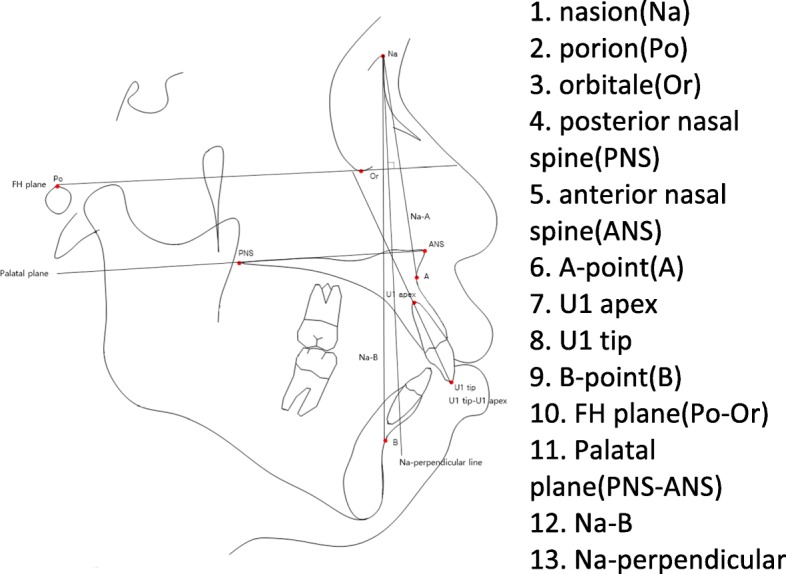
Landmarks and reference lines used in cephalometric analysis

**Fig. 2:**
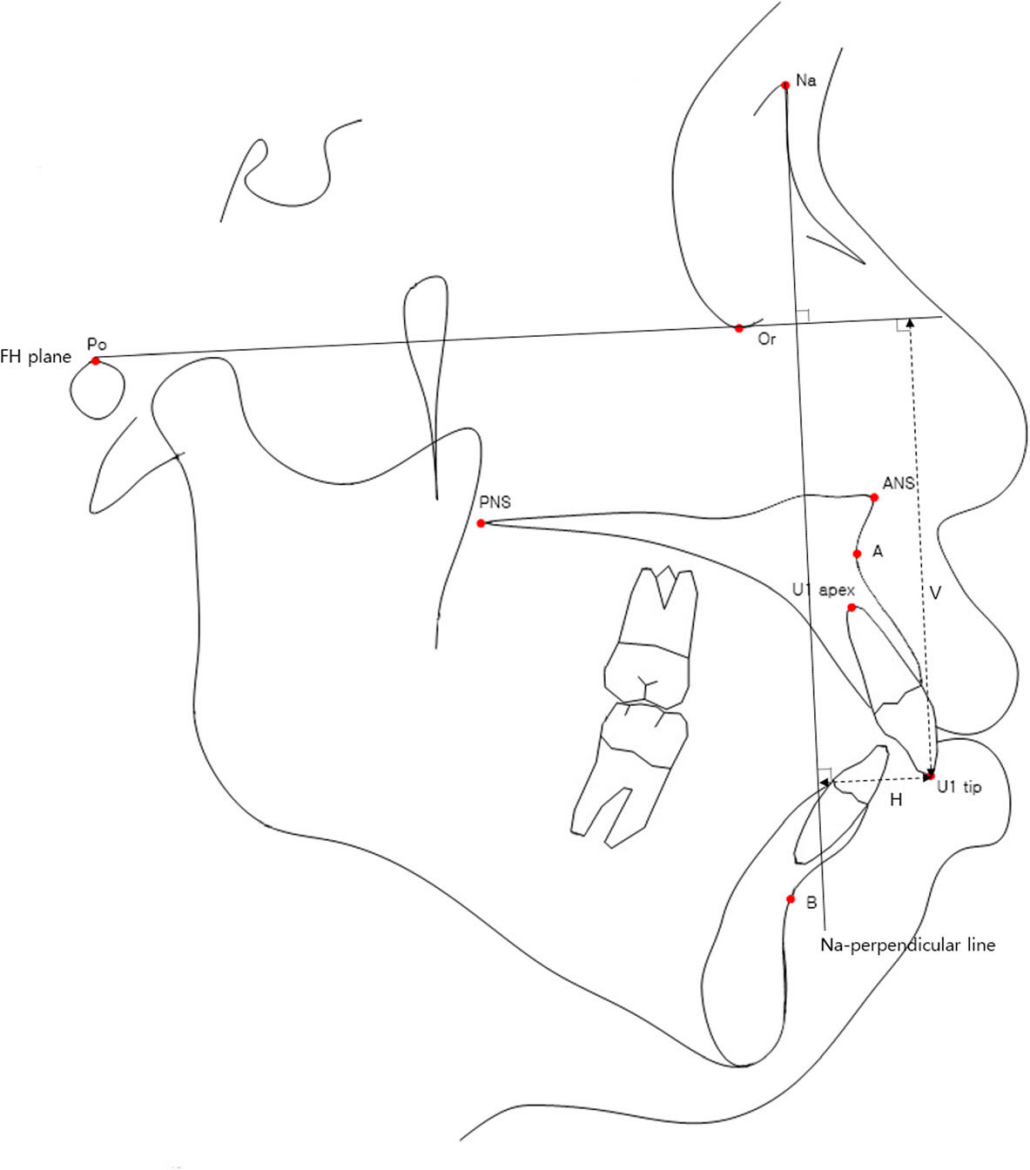
Reference lines used to determine the extent of intrusion and retraction of the incisor. Difference in the distance from incisor tip to Frankfort plane at pre-treatment and post-treatment (V0–V1) represents the amount of intrusion of incisor. Difference in the distance from incisor tip to N-perpendicular line at pretreatment and posttreatment (H0–H1) represents the amount of retraction of incisor

**Fig. 3:**
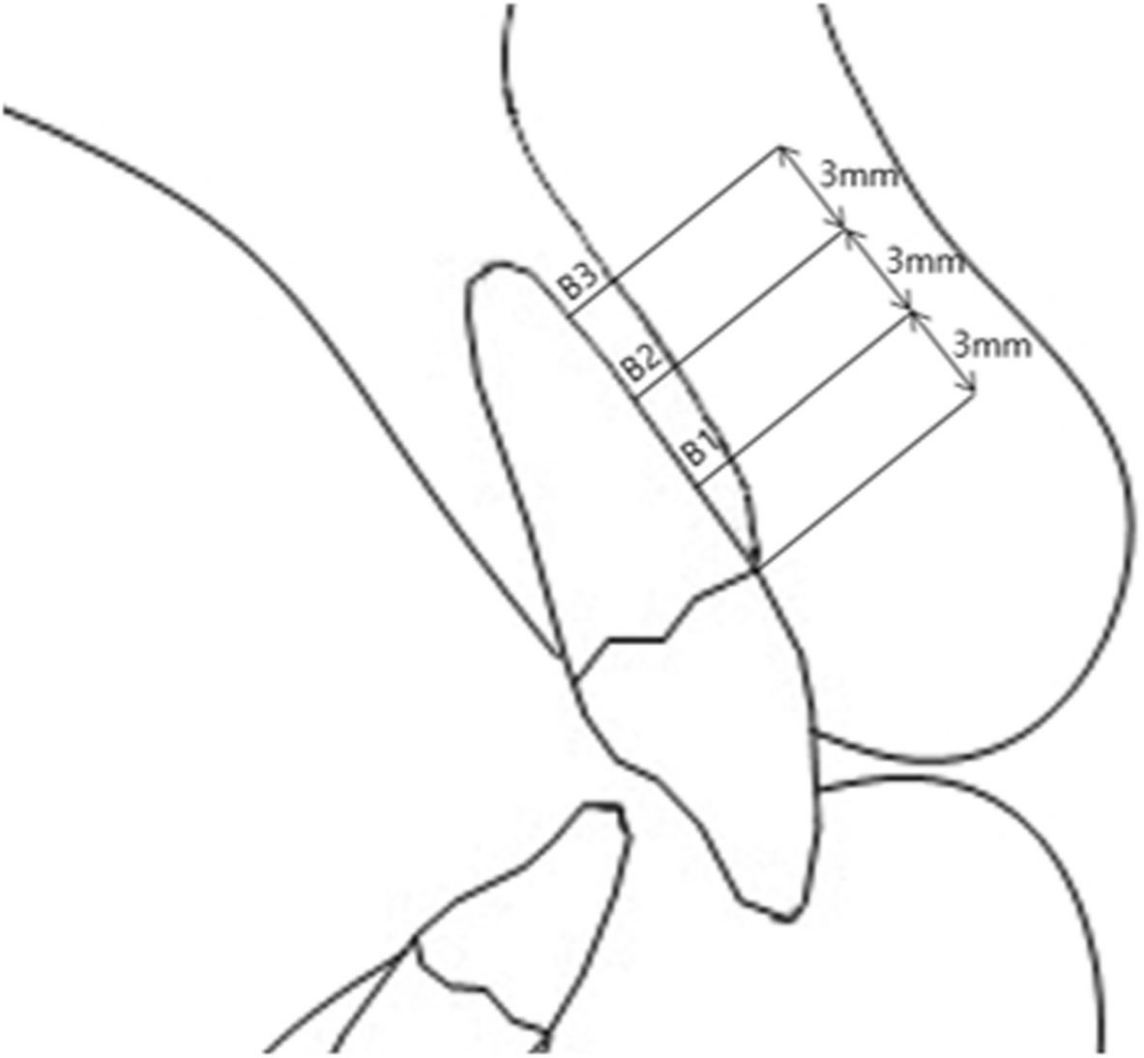
Measured thickness between the incisor and surrounding bone. Distance between incisor and alveolar bone measured at 3-mm intervals from alveolar crest level

**Fig. 4:**
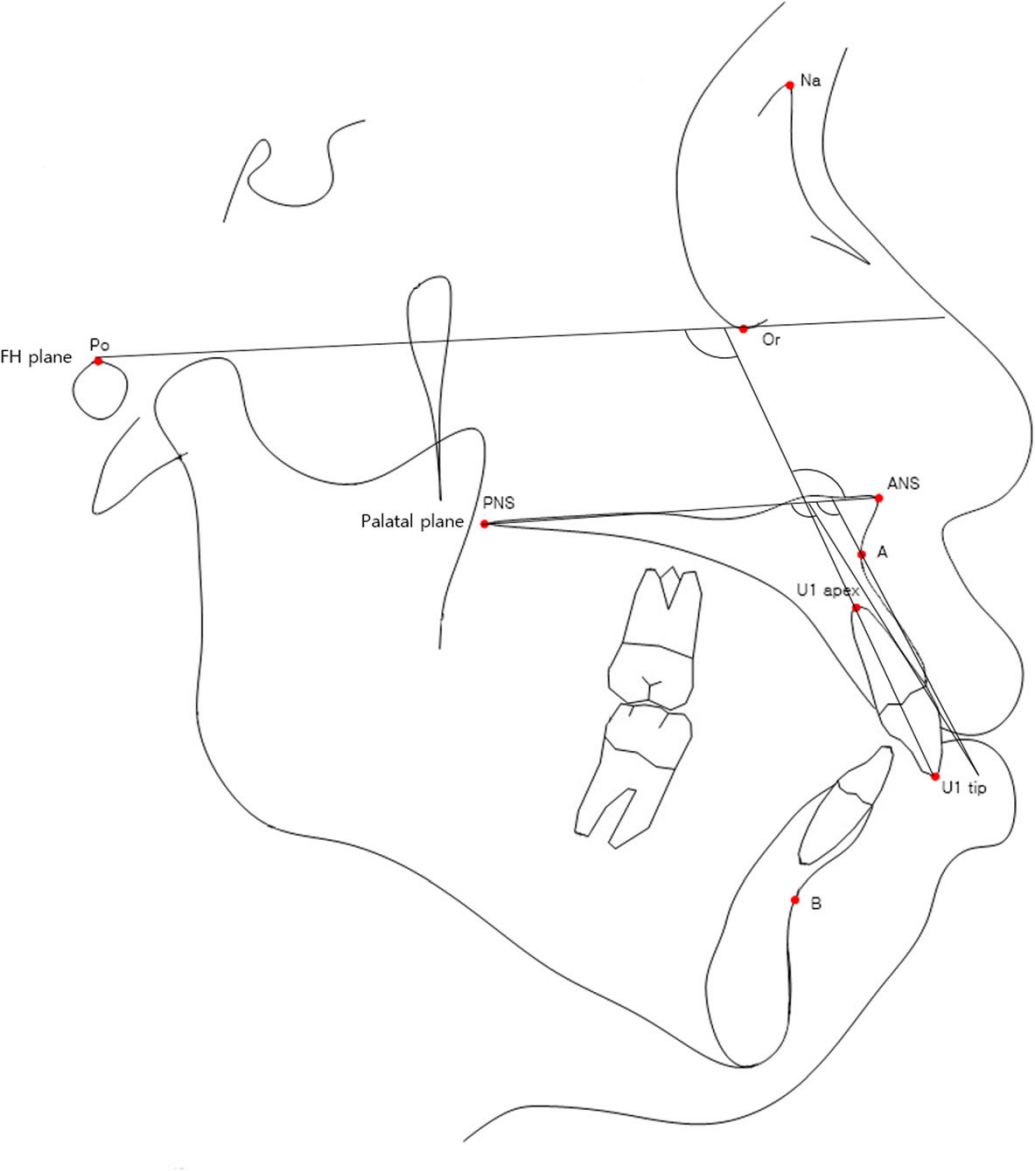
Angular variables used to determine the extent of intrusion and retraction of the incisor

**Fig. 5:**
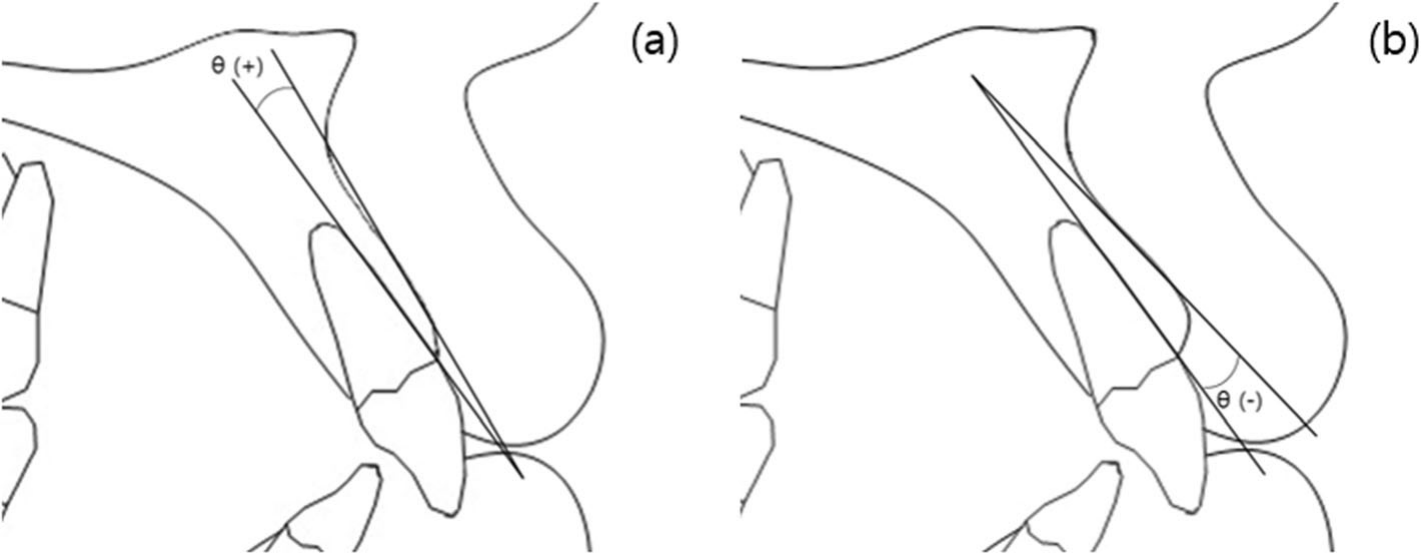
Measured angle (**a**, **b**) between the incisor and surrounding bone. **a**, **b** The angle between the line touching the margin of the tooth at the alveolar crest level and the line touching the marginal margin of the alveolar bone

Pre-treatment and post-treatment lateral cephalometric radiographs were taken, and a total of 13 linear, angular variables of facial structures which could affect the alveolar bone remodeling of upper incisors were measured (Figs. 1 and 2). U1 tip to N-perpendicular line (mm) was measured to calculate the amount of retraction of the incisor, and U1 tip to Frankfort horizontal plane (mm) was also measured to calculate the amount of intrusion of the incisor (Fig. 2). The angle of the incisor axis to Frankfort horizontal plane, the angle of the incisor axis to palatal plane, the angle of the incisor surface to palatal plane, and the angle of the alveolar bone surface to palatal plane were measured (Fig. 3). Distance between incisor and labial alveolar bone was measured at three levels at both pre-treatment (T0) and post-treatment (T1) radiographs. The distances at the levels were categorized as B1, B2, and B3 according to 3-, 6-, and 9-mm distance from the cementoenamel junction. The distance was measured while the picture was magnified 3 times for accuracy followed by shrinking back to the original 1:1 scale. Measurements were taken at the outermost point (Fig. 4). The angle between the line most tangent to the margin of the incisor at the alveolar crest level and the line most tangent to the outline of the alveolar bone (*θ*) was measured at both stages. The most tangent line was drawn with the following sequence. First, FH to Nasion perpendicular line was established as a vertical reference line. The closest point from the line to the alveolar bone or the maxillary central incisor was found as an original point. Next, A protractor was used to find the tangent point. The point where the protractor first meets the superior structure from the origin is the tangent point. The tangent line was composed of those two points. When two lines meet in the direction of the incisor tip, the angle was defined as positive, and when the lines meet at the side of the apex, the angle was defined as negative (Fig. 5a, b). All measurements were performed twice by a single investigator at 4-week intervals. Paired *t* tests at a 0.05 significance level were used to evaluate the movement of upper central incisors and the changes of thickness and angle between incisors and labial alveolar bone, as an index of the alveolar bone remodeling after retraction and intrusion of incisors. Pearson’s correlation coefficients were measured to identify variables related to alveolar bone remodeling with a significance level of 0.05.

## Results

Measurement error was estimated for two sets of data using Dahlberg’s formula [12]. Although the Paired *t* test indicated a statistically significant increase of labial alveolar bone thickness at 9-mm distance from cementoenamel junction (B3) (*P* < 0.05), the clinical meaning of 0.4-mm apposition seems minute. No statistically significant change in the thickness at other levels or the angle between the alveolar bone and the incisor (Table 1). Individual changes had significant variance from the statistical mean. Of the 34 patients, 16 had increased B1, 17 had decreased B1, and 1 had no change in B1. Seventeen had increased B2, 14 had decreased B2, and 3 remained unchanged. B3 stayed the same for one patient, and all others had increased B3. The angle *θ* increased in 21 patients and decreased in 13 patients.

**Table 1:** Comparison of mean labial alveolar bone thickness and angulation at initial and final stage

	Initial stage		Final stage		
	Mean	SD	Mean	SD	*P* value
Thickness					
B1	1.06	0.409	1.19	0.776	0.358
B2	1.16	0.449	1.40	0.905	0.140
B3	1.56	0.647	1.95	1.094	0.032*
Angulation					
*θ*	1.18	4.710	3.77	6.148	0.058

B1, B2, B3: labial alveolar bone thickness at 3-, 6-, and 9-mm distance from the alveolar crest; *θ*: measured angle between the line tangent to the margin of the incisor at the alveolar crest level and the line tangent to the outline of the alveolar bone

*SD*, standard deviation

*P < 0.05, **P < 0.005, ***P < 0.001, statistically significant; paired *t* test

ΔB/initial B at each level was calculated to find the relative change of thickness compared with the initial state. ΔB1/initial B1, ΔB2/initial B2, and ΔB3/initial B3 are correlated with the initial B1, initial B2, and initial B3, respectively. The amounts of B1 change, B2 change, and B3 change (ΔB1, ΔB2, ΔB3) are correlated with the initial palatal plane to upper incisor angle, the initial palatal plane to upper incisor labial surface angle, and the initial palatal plane to bone labial surface angle. ΔB3/initial B3 is correlated with the initial *θ*. ΔB1/initial B1 and ΔB3/initial B3 are correlated with the initial palatal plane to upper incisor angle, the initial palatal plane to upper incisor labial surface angle, and the initial palatal plane to bone labial surface angle, while ΔB2/initial B2 is not significantly correlated. ΔB1 and ΔB3 are correlated with U1 to FH. ΔB1, ΔB2, and ΔB3 are correlated with each other. ΔB1/initial B1, ΔB2/initial B2, and ΔB3/initial B3 are also correlated with each other. ΔB3 and ΔB3/initial B3 are correlated with the intrusion amount. It was found that change was not related to the retraction amount (Table 2). The initial angulation between the labial surface and corresponding alveolar bone of upper incisors are correlated with the amount of the angulation change (Table 3).

**Table 2:** Correlation between mean changes of bone thickness and potential influencing factors; *r* value (*P* value)

	ΔB1	ΔB1/B1 at T0	ΔB2	ΔB2/B2 at T0	ΔB3	ΔB3/B3 at T0
B1 at T0	− 0.288 (0.099)	− 0.341* (0.048)	− 0.183 (0.301)	− 0.286 (0.102)	0.093 (0.601)	− 0.075 (0.674)
B2 at T0	− 0.214 (0.225)	− 0.333 (0.055)	− 0.327 (0.059)	− 0.435* (0.010)	− 0.085 (0.631)	− 0.281 (0.107)
B3 at T0	− 0.051 (0.775)	− 0.144 (0.417)	− 0.152 (0.391)	− 0.238 (0.176)	− 0.196 (0.268)	− 0.403* (0.018)
*θ* at T0	− 0.078 (0.662)	− 0.163 (0.357)	− 0.139 (0.433)	− 0.194 (0.270)	− 0.324 (0.062)	− 0372* (0.030)
U1 to FH	− 0.386* (0.024)	− 0.303 (0.081)	− 0.279 (0.111)	− 0.191 (0.280)	− 0.359* (0.037)	− 0.265 (0.130)
U1 to Palatal plane	− 0.428* (0.011)	− 0.339* (0.050)	− 0.428* (0.012)	− 0.248 (0.157)	− 0.448* (0.008)	− 0.354* (0.040)
U1 labial surface to palatal plane	− 0.522** (0.002)	− 0.395* (0.021)	− 0.372* (0.030)	− 0.239 (0.174)	− 0.432* (0.011)	− 0.389* (0.023)
Bone labial surface to palatal plane	− 0.479** (0.004)	− 0.408* (0.017)	− 0.366* (0.033)	− 0.314 (0.070)	− 0.405* (0.018)	− 0.404* (0.018)
ΔB1	1	0.917*** (< 0.001)	0.838*** (< 0.001)	0.742*** (< 0.001)	0.667*** (< 0.001)	0.617*** (< 0.001)
ΔB1/B1 at T0	0.917*** (< 0.001)	1	0.861*** (< 0.001)	0.873*** (< 0.001)	0.689*** (< 0.001)	0.741*** (< 0.001)
ΔB2	0.838*** (< 0.001)	0.861*** (< 0.001)	1	0.938*** (< 0.001)	0.818*** (< 0.001)	0.771*** (< 0.001)
ΔB2/B2 at T0	0.742*** (< 0.001)	0.873*** (< 0.001)	0.938*** (< 0.001)	1	0.762*** (< 0.001)	0.832*** (< 0.001)
ΔB3	0.667*** (< 0.001)	0.689*** (< 0.001)	0.818*** (< 0.001)	0.762*** (< 0.001)	1	0.875*** (< 0.001)
ΔB3/B3 at T0	0.617*** (< 0.001)	0.741*** (< 0.001)	0.771*** (< 0.001)	0.832*** (< 0.001)	0.875*** (< 0.001)	1
Intrusion amount	0.243 (0.166)	0.223 (0.205)	0.186 (0.292)	0.213 (0.226)	0.392* (0.022)	0.386* (0.024)
Retraction amount	0.155 (0.380)	0.128 (0.472)	− 0.004 (0.983)	0.005 (0.978)	− 0.185 (0.294)	− 0.125 (0.483)

*P < 0.05, **P < 0.005, ***P < 0.001, statistically significant; Pearson’s correlation coefficient

**Table 3:** Correlation between mean changes of the angle and potential influencing factors; *r* value (*P* value)

	Δ*θ*
B1 at T0	0.144 (0.417)
B2 at T0	0.049 (0.784)
B3 at T0	− 0.047 (0.790)
*θ* at T0	− 0.602*** (< 0.001)
U1 to FH	− 0.060 (0.737)
U1 to palatal plane	− 0.099 (0.579)
U1 labial surface to palatal plane	− 0.134 (0.451)
Bone labial surface to palatal plane	− 0.073 (0.680)
Intrusion amount	0.110 (0.537)
Retraction amount	− 0.317 (0.068)

*P < 0.05, **P < 0.005, ***P < 0.001, statistically significant; Pearson’s correlation coefficients

## Discussion

A systematic review reported that increased labial bone thickness at the cervical third level was found during the en-masse retraction of the anterior teeth [13]. In the present study, the alveolar bone contour change during upper incisor intrusion and retraction was evaluated.

The alveolar bone surrounding the maxillary incisors does not always follow the tooth movement during upper incisor intrusion and retraction. The unpredictability of the direction and amount of movement in alveolar bone remodeling poses a serious challenge to the treatment result. The aim of this study was to determine the initial factors that would affect alveolar bone remodeling. We analyzed the common variables and determined which ones would have predictive values before treatment.

The variability of outcome was noted by a few authors. For example, Ren’s systematic literature review [14] suggested to conduct more clinical studies and experiments to gain more insight on optimal applied force and the rate of movement in orthodontics. Felicita [15] found that there are different clinical outcomes depending on the positions of anchors and length of attachment. In addition, Yodthong’s study [16] believed that some factors, such as rate of tooth movement, change in inclination, and extent of intrusion may influence the alveolar bone thickness during upper incisor retraction.

**Fig. 6:**
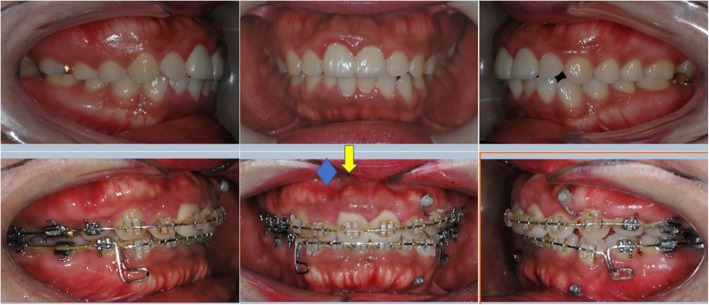
Esthetically compromised alveolar bone irregularity are shown during incisors intrusion and retraction. Arrow indicated insufficient alveolar bone remodeling under maxillary central incisor involves from B1 to B3 level, while diamond indicated that only B3 level irregularity is shown

This study found that the changes in alveolar bone thickness at levels B1, B2, and B3 are intercorrelated, which means once a tooth presented with little or no alveolar bone resorption, all the B1 to B3 three levels showed the same tendency. This phenomenon is frequently observed (Fig. 6) and demonstrated the results of Table 2.

The change in angulation between the incisor and alveolar bone was also negatively correlated with the initial angulation.

The change in labial bone thickness was not significantly correlated with the initial thickness, while the relative change, calculated as ΔB/B at T0, showed a negative correlation with the initial thickness.

This study found that there was only correlation between the amount of intrusion to the change in labial alveolar bone at B3 level, not B1 or B2. Also, there was no significant correlation between the amount of retraction to the change in labial alveolar bone thickness. The result from this study was different from Pornputti’s results [17], which claimed that tooth movement and the change in labial alveolar bone are statistically correlated, and Atik’s results [18], which claimed to be unrelated. Different mechanics including anchorage position and traction vectors might influence the different values.

In this study, we have also shown that the inclination of upper incisors and alveolar bone were correlated to the remodeling of alveolar bone. When upper incisors or the alveolar bone was proclined, the ΔB1, ΔB2, and ΔB3 had low values, which means resorption than addition.

**Fig. 7:**
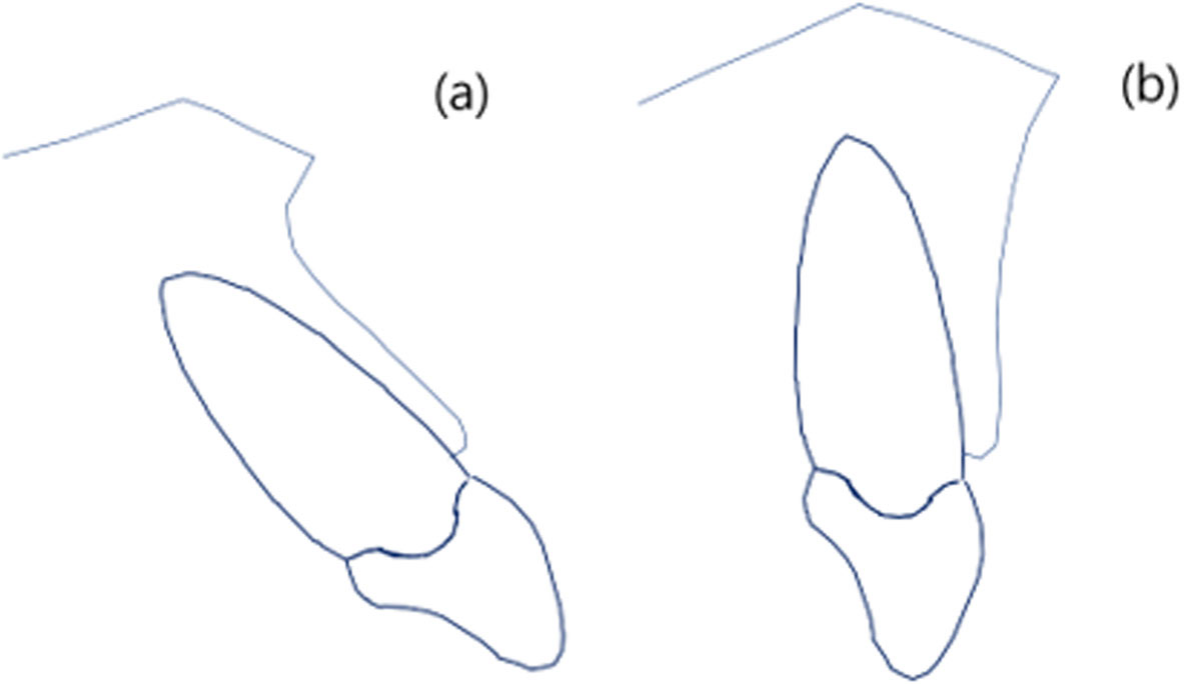
The condition describes alveolar bone architecture tends **a** to follow as the incisors retracted **b** not to be remodeled as expected in Fig. 6

In orthodontic treatment cases, initial conditions such as FH plane to upper incisor angle, palatal plane to upper incisor angle, and palatal plane to labial surface of upper incisor angle should be carefully assessed before proceeding with treatment to better predict the outcome of treatment (demonstrated in Fig. 7). Changes in alveolar bone thickness may produce undesirable esthetics and other side effects.

There are a few limitations and areas of improvements for this study. Measurements were taken with lateral cephalometric radiographs, further quantitative research based on three-dimensional computed tomography will greatly improve the higher resolution of images and finer measurements. A longitudinal observation in addition to the starting and ending points might provide more detail on how continuous modeling occurs.

## Conclusion

Factors associated with alveolar bone remodeling included the angle of upper incisor to FH plane, anterior alveolar bone to FH plane. The relative change of alveolar bone thickness, calculated as ΔB/B at T0, showed a negative correlation with the initial thickness. Changes in the angle between the labial surface of upper incisor and alveolar bone also correlated with the initial contour of alveolar bone. The amount of retraction does not affect the remodeling of alveolar bone. The suggested factors related to alveolar bone remodeling in this study would enhance the predictability of alveolar bone response in patients with upper incisor intrusion and retraction, and provide an insight into safe and reliable orthodontic treatment. Further quantitative research based on three-dimensional computed tomography will be helpful to reveal more details.

## Data Availability

The data supporting the study can be obtained directly from the authors.

## Abbreviations

": Inches
ANB: Angle between the lines NA and NB
B1: 3-mm distance from the cementoenamel junction
B2: 6-mm distance from the cementoenamel junction
B3: 9-mm distance from the cementoenamel junction
IRB: Institutional review board
mm: Millimeter
P: Probability value
T0: Pre-treatment
T1: Post-treatment
TADs: Temporary anchorage devices
U1 to N-A: Linear measure between the most vestibular point of the upper incisor and the NA line
*Δ*: Delta, change in quantity that can be changed
*θ*: The angle between the line most tangent to the margin of the incisor at the alveolar crest level and the line most tangent to the outline of the alveolar bone
